# Design and introduction of a quality of life assessment and practice support system: perspectives from palliative care settings

**DOI:** 10.1186/s41687-018-0065-2

**Published:** 2018-08-22

**Authors:** Richard Sawatzky, Esther Laforest, Kara Schick-Makaroff, Kelli Stajduhar, Sheryl Reimer-Kirkham, Marian Krawczyk, Joakim Öhlén, Barbara McLeod, Neil Hilliard, Carolyn Tayler, S. Robin Cohen

**Affiliations:** 1 School of Nursing, Trinity Western University, 7600 Glover Road, Langley, BC V2Y 1Y1 Canada; 2 Centre for Health Evaluation and Outcome Sciences, Providence Health Care, 588 – 1081 Burrard Street, St. Paul’s Hospital, Vancouver, BC V6Z 1Y6 Canada; 3 Ingram School of Nursing, McGill University, 680 Sherbrooke Street West, Montreal, QC H3A 2M7 Canada; 4 Faculty of Nursing, University of Alberta, Level 3, Edmonton Clinic Health Academy, 11405-87 Avenue, Edmonton, AB T6G 1C9 Canada; 5 School of Nursing and Institute on Aging & Lifelong Health, (IALH), University of Victoria, PO Box 1700, STN CSC, Victoria, BC V8W 2Y2 Canada; 6 Palliative Care, Fraser Health, 100 – 2296 McCallum Road, Abbotsford, BC V2S 3P4 Canada; 7 Faculty of Graduate Studies, University of Calgary, MacKimmie Tower, Room 213, 2500 University Drive NW, Calgary, AB T2N 1N4 Canada; 8 Faculty of Graduate Studies, University of Victoria, PO Box 3025 STN CSC, Victoria, BC V8W 3P2 Canada; 9 Center for Person-Centered Care, Institute of Health and Care Sciences, Salgrenska Academy, University of Gothenburg, Box 457, 405 30 Göteborg, Sweden; 10 BC Centre for Palliative Care, 300 – 601 Sixth St., New Westminster, BC V3L 3C1 Canada; 11 Departments of Oncology and Medicine, McGill University, Montreal, QC, Canada; 12 Lady Davis Research Institute of the Jewish General Hospital, 3755 Côte Ste. Catherine Road, Montreal, QC H3T 1E2 Canada; 13 Salgrenska Academy, University of Gothenburg, Box 457, 405 30 Göteborg, Sweden; 14 Palliative Centre, Sahlgrenska University Hospital, Box 30110, 400 43 Göteborg, Sweden

**Keywords:** Quality of life assessment, Palliative care, Person-centered care, Older adults, Family caregivers

## Abstract

**Background:**

Quality of life (QOL) assessment instruments, including patient-reported outcome measures (PROMs) and patient-reported experience measures (PREMs), are increasingly promoted as a means of enabling clinicians to enhance person-centered care. However, integration of these instruments into palliative care clinical practice has been inconsistent. This study focused on the design of an electronic Quality of Life and Practice Support System (QPSS) prototype and its initial use in palliative inpatient and home care settings. Our objectives were to ascertain desired features of a QPSS prototype and the experiences of clinicians, patients, and family caregivers in regard to the initial introduction of a QPSS in palliative care, interpreting them in context.

**Methods:**

We applied an integrated knowledge translation approach in two stages by engaging a total of 71 clinicians, 18 patients, and 17 family caregivers in palliative inpatient and home care settings. Data for Stage I were collected via 12 focus groups with clinicians to ascertain desirable features of a QPSS. Stage II involved 5 focus groups and 24 interviews with clinicians and 35 interviews with patients or family caregivers during initial implementation of a QPSS. The focus groups and interviews were recorded, transcribed, and analyzed using the qualitative methodology of interpretive description.

**Results:**

Desirable features focused on hardware (lightweight, durable, and easy to disinfect), software (simple, user-friendly interface, multi-linguistic, integration with e-health systems), and choice of assessment instruments that would facilitate a holistic assessment. Although patient and family caregiver participants were predominantly enthusiastic, clinicians expressed a mixture of enthusiasm, receptivity, and concern regarding the use of a QPSS. The analyses revealed important contextual considerations, including: (a) logistical, technical, and aesthetic considerations regarding the QPSS as a technology, (b) diversity in knowledge, skills, and attitudes of clinicians, patients, and family caregivers regarding the integration of electronic QOL assessments in care, and (c) the need to understand organizational context and priorities in using QOL assessment data.

**Conclusion:**

The process of designing and integrating a QPSS in palliative care for patients with life-limiting conditions and their family caregivers is complex and requires extensive consultation with clinicians, administrators, patients, and family caregivers to inform successful implementation.

## Background

People living with life-limiting chronic conditions (such as chronic cardiovascular and respiratory conditions, cancer, diabetes and complications thereof, and degenerative neurological disorders) often have multifaceted health care needs related to the burden of complex symptoms and the impacts of advancing illness on their quality of life (QOL). A palliative approach to care is recommended to address these needs from a person-centered point of view early on in the disease trajectory [1–3]. As defined by the World Health Organization, “palliative care is an approach that improves the QOL of patients and their families facing the problems associated with life-threatening illness, through the prevention and relief of suffering by means of early identification and impeccable assessment and treatment of pain and other problems, physical, psychosocial, and spiritual” [4]. QOL assessment instruments, including patient-reported outcome measures (PROMs) and patient-reported experiences measures (PREMs) [5–7], are increasingly promoted as a means of enabling clinicians to efficiently assess and respond to aspects of health and healthcare that are relevant to the QOL of patients and family caregivers from their point of view [8–13]. Primary studies and systematic reviews suggest that providing clinicians with this kind of information can improve patient-clinician communication, raise awareness of problems that would otherwise be unidentified, improve care plans, and improve multidisciplinary collaboration [8, 10, 14–26]. In addition, health care administrators and managers increasingly advocate for the routine use of PROMs and PREMs because of their potential to enhance person-centered care by ensuring that the perspectives and experiences of patients and family caregivers are revealed and integrated in point-of-care and managerial decision making.

Despite evidence supporting the benefits of QOL assessments and the availability of many QOL assessment instruments for palliative care populations [27], the integration of these instruments into palliative care clinical practice has been elusive [8, 28]. Shortcomings of previous studies on integrating PROMs into practice include: 1) a lack of consultation with clinicians about the design of QOL assessment instruments, 2) a paucity of information about how to integrate them into practice, as well as 3) clinicians not wanting to measure outcomes they feel ill-prepared to address [8, 14, 19, 20, 23]. To promote use in routine clinical practice, it is critical that QOL assessment instruments can be smoothly integrated into the practice of clinicians and that the information can be summarized to provide on-the-spot, easily interpretable reports regarding priority areas of concern for patients and family caregivers, and corresponding recommendations for practice.

User-centered electronic information systems, made accessible at the point of care, are recommended to facilitate the integration of QOL assessment instruments in practice [13, 24, 29–36]. The electronic format enables the integration of the results of patients’ and family caregivers’ QOL assessments directly into the patients’ charts or electronic medical records [31]. Various electronic assessment systems have been studied with a focus on particular aspects of care [30–32, 37–40], such as symptom management (e.g., *Edmonton Symptom Assessment System*) [37]. However, studies of people with life-limiting illnesses have predominantly focused on oncology patients, and there has been little emphasis on family caregivers’ QOL and experiences with the care provided (PREMs) [28, 41]. There is a paucity of knowledge about the perspectives of clinicians, patients, and family caregivers regarding the desirable characteristics and integration of electronic QOL assessment systems in palliative care settings. For the integration of such electronic systems to be successful, it is imperative that the perspectives of all end users (e.g., clinicians, patients, family caregivers) are known and used to inform their design, development, implementation, and evaluation [8, 42–44].

### Aim

This study is part of an overarching research initiative on the development and integration of an electronic Quality of Life and Practice Support System (QPSS) for the use of PROMs and PREMs in clinical practice. The current study focused on the user-centered design of a QPSS prototype and its initial use in palliative inpatient and home care settings. Our objectives were to: (a) ascertain clinicians’ desired features of a QPSS prototype and (b) elicit the experiences of clinicians, patients, and family caregivers in regard to the introduction of a QPSS prototype in palliative care.

## Methods

The study had two sequential stages based on the initial phases of the Knowledge To Action (KTA) cycle [45, 46] to inform the user-centered design and initial use of a QPSS prototype. The KTA cycle emphasizes an action-oriented approach to engaging different knowledge users (in this case, clinicians, administrators, patients, and family caregivers) in the process of creating, contextualizing, and implementing knowledge in a particular practice setting. Stage I involved an exploration of clinicians’ desired features of a QPSS as the basis for its design and integration in clinical practice. This included preferences regarding the choice of QOL assessment instruments (PROMs and PREMs) as well as practical usability features of both the software and the hardware. Stage II involved obtaining formative evaluative feedback on initial use of a QPSS prototype from clinicians, patients, and family caregivers, with a focus on assessing contextual considerations that may impact routine integration of QOL assessments in clinical practice.

### A quality of life and practice support system (QPSS) prototype

A QPSS prototype was developed within the context of this project by Intogrey Research and Development Inc. as an electronic handheld tablet-based system to facilitate QOL assessments in daily clinical practice. Clinicians, administrators, and researchers participating in this project contributed to the original design and ongoing development of the QPSS prototype throughout the study. The goal was to develop a person-centered health care information system that facilitates the use of standardized QOL assessment instruments at the point of care via tablets to assess patients’ and their family caregivers’ QOL concerns and health care experiences. The QPSS prototype was specifically designed to incorporate different QOL assessment instruments (PROMs and PREMs) that are appropriate for patient and family caregiver populations while addressing both clinical and administrative needs. In stage 2 of the study, patients and family caregivers used the QPSS to independently enter responses to the assessment questions. Clinicians could also enter responses as part of an assessment interview (but not as a proxy). The system could produce instantaneously scored information and reports in both customizable tabular and graphical formats. Additional features developed in response to project findings are described in the Results section.

### Settings and samples

The study was conducted primarily at two sites: a palliative home care setting and an inpatient palliative care unit located in suburbs near Vancouver (British Columbia, Canada). The palliative home care team included seven nurses who had enhanced training in end of life care and who, in close collaboration with the rest of the palliative care team and the family doctor, provided ongoing support at home for patients with life-limiting illness and their family caregivers. The inpatient palliative care unit staff consisted of regular and casually-employed nurses, a patient care coordinator, a unit clerk, a social worker, a pharmacist, and several physicians who provide care for palliative care patients with acute symptom management needs. All clinicians (including those in administrative or managerial positions) and patients who were 50 years or older and receiving palliative care were eligible to participate. Family caregivers were identified by the competent patient or the clinician as one of the people most involved with the patient. The patients and family caregivers had to speak and read English and be capable of and willing to provide written informed consent. Clinicians, including those with administrative or managerial responsibilities, were recruited during team meetings at each primary site when the project was presented, as well as via emailed invitations. Clinicians were asked to approach patients as part of routine care to elicit patients’ interest in meeting with a research team member, who subsequently followed up to explain the study and invite participation. Family caregivers were approached at the same time or during a follow-up meeting.

For the purposes of obtaining the perspectives of clinicians working in different jurisdictions, clinician focus groups were run in five other home health offices. Three were in a different health care system, in Montreal in the province of Quebec (healthcare in Canada is a provincial jurisdiction), of which one involved an interdisciplinary team as part of a public local community health center, and two were private not-for-profit palliative nursing care teams that received referrals from the public health care system and self-referrals from patients and family caregivers. Two additional public home health offices were in British Columbia in rural jurisdictions outside of the metropolitan greater-Vancouver area and part of a different health authority. Clinicians at these additional sites were recruited via emailed invitations, either directly to them or to the service director who then informed the staff of the research opportunity. All participants provided written informed consent. Study protocols were reviewed and approval was granted by research ethics boards of applicable academic and health care organizations.

The resulting samples across the two primary sites included 46 clinician participants, 18 patients, and 17 family caregivers. In addition, there were 25 clinicians in focus groups at the five other home health offices. The clinician participants were predominantly female, with ages ranging from 23 to 63 years. Most clinicians were registered nurses; others were medical doctors, social workers, spiritual care workers, allied health professionals, and licensed practical nurses. Seven clinician participants had responsibilities in health care administration. The patient participants were predominantly male, with ages ranging from 46 to 95 years. The family caregivers were predominantly female, with ages ranging from 51 to 89 years. Detailed participant characteristics for the two primary sites are provided in Table 1.

**Table 1:** Demographic information of participants in individual interviews and focus groups

	Palliative Home Care				Palliative Inpatient Care		
	Patients	Family caregivers	Clinicians at primary site	Clinicians at other sites	Patients	Family caregivers	Clinicians
Sample size	15	12	21	25	3	5	25
Age							
Median	73	68	45	49	61	57	43
Range (min - max)	46–95	56–89	34–61	35–60	58–66	51–62	23–63
Missing (%)	0	1(8%)	3(14%)	1(4%)	0	0	3(12%)
Gender							
Male	12	3	0	3	1	1	4
Female	3	9	19	22	2	4	20
Missing (%)	0	0	2(10%)	0	0	0	1(4%)
Highest education Level							
Less than High School	1	0	0	0	0	0	0
High School	4	6	0	0	0	1	0
College/Diploma	4	2	5	5	1	2	1
University (Bachelor)	4	2	8	15	1	1	16
Graduate	2	1	6	5	1	1	7
Missing (%)	0	1(8%)	2(10%)	0	0	0	1(4%)
Occupation							
Medical doctor	–	–	2	0	–	–	2
Registered Nurse	–	–	16	21	–	–	19
Licensed Practical Nurse	–	–	1	1	–	–	0
Other	–	–	1	3	–	–	3
Missing (%)	–	–	1(5%)	0	–	–	1(4%)
Years in position							
Median	–	–	6	8	–	–	8
Range (min - max)			1–15	1–26			1–26
Missing (%)			1(5%)	0			1(4%)
Position							
Casual	–	–	1	3	–	–	3
Permanent	–	–	14	19	–	–	19
Administration	–	–	5	3	–	–	1
Missing	–	–	1(5%)	0	–	–	2(8%)
Country/continent of birth							
Canada	11	7	15	22	1	3	18
Asia	0	0	2	0	0	0	0
Europe	4	3	0	2	0	1	3
Other	0	1	2	1	0	1	3
Missing (%)	0%	1(8%)	2(10%)	0	2(67%)	0%	1(4%)

### Data collection

Data for Stage I of the project were collected via seven focus groups with clinicians at both primary study sites (two at the palliative home care setting and five at the palliative care unit) and five focus groups with clinicians at the additional home health offices (one focus group at each office). The focus groups were guided by questions regarding opinions about and previous experience with using QOL assessment instruments, information to be gathered via QOL assessments, and desirable features of an electronic QPSS. Based on initial focus group results and a literature review of QOL assessment instruments, participants were presented with candidate QOL assessment instruments for their consideration, which, once agreed upon, were programmed into the prototype QPSS. Data collection during Stage II of the project included additional focus groups at the primary study sites (four at the palliative home care setting and one at the palliative care unit), as well as 24 interviews with clinicians, 3 with patients, and 5 with family caregivers at the palliative care unit, and 15 interviews with patients and 12 with family caregivers at the home care setting (there were no individual interviews with clinicians at the home care setting). During Stage II, clinicians had opportunity to try the QPSS prototype as part of the focus groups and during home care visits with consenting patients and family caregivers. Clinicians selected which of the available instruments each patient and caregiver completed (i.e., completing all instruments was not required). In addition, the QPSS prototype was used by patients and family caregivers with research assistants, prior to being interviewed. The Stage II focus groups and interviews were guided by questions about experiences with using QOL assessment instruments and the QPSS, as well as questions about additional desirable features. In addition, information about the frequency of use and duration to complete of each QOL assessment instrument was collected directly via the QPSS prototype.

### Data analysis

The focus groups and interviews were recorded digitally, transcribed verbatim, and analyzed using the NVIVO™ software and guided by the qualitative methodology of interpretive description [47]. Preliminary summaries of focus groups were developed on an ongoing basis and used to inform the QPSS prototype design and subsequent focus groups. Initial coding of the transcripts was completed by three research team members (KSM, EL, MK) with ongoing supervision provided RS and RC. Transcripts were read and re-read to generate initial codes, which were then discussed and refined in an iterative fashion through group coding and rounds of feedback with members of the research team. Final thematic analyses involved evolving levels of abstraction to identify central themes. For example, initial codes for Stage I (prior to QPSS use) focused on opinions and perspectives regarding the selection of QOL assessment instruments, desired features of a QPSS, and facilitators and barriers regarding the integration of a QPSS in practice. These were elaborated upon during the subsequent analysis of Stage II data based on the experiences of clinicians, patients, and family caregivers following initial introduction of the QPSS prototype. In addition, coding of Stage II data focused on contextual considerations for introducing a QPSS in clinical practice. These initial codes were subsequently interpreted and organized to construct patterns regarding different types of features and contextual considerations pertaining to use of the QPSS prototype. Well-established recommendations were followed to ensure trustworthiness and rigor, including (a) methods to address credibility (iterative cycles of engagement with clinicians, patients, caregivers, and multiple sources of data), (b) confirmability (audit trail), and (c) transferability (detailed reporting of the context) to enable transferring the findings to other contexts [47, 48].

## Results

During Stage I, clinicians demonstrated significant interest in having opportunity to inform the ongoing design and adaptation of a QPSS prototype. Many desired features that would facilitate use of the QPSS in routine clinical practice were discussed. Additional features were discussed by clinicians, patients, and family caregivers following initial trialing of a QPSS prototype in Stage II.

### Desired features of the QPSS

Desired features of the QPSS included assessment instruments selected for use, as well as hardware and software programming considerations. Clinicians recognized the importance of selecting QOL assessment instruments that would facilitate a holistic assessment of physical, psychological, social, and even existential aspects of life. *The Edmonton Symptom Assessment System - Revised (ESAS-r)* [39] instrument was selected, in part because hospital clinicians were already using this instrument in paper form. Not surprisingly, the ESAS-*r* was also the most extensively used instrument during Stage II of the project. However, clinicians and administrators also expressed the need to include more comprehensive patient and family caregiver QOL assessment instruments, leading to selection of the *McGill Quality of Life Questionnaire - Revised (MQOL-R*) [49] for patients and the *Quality of Life in Life Threatening Illness--Family Carer Version (QOLLTI-F)* [50] for family caregivers. Administrators and practice leaders additionally emphasized assessment of patients’ and family caregivers’ satisfaction and experiences with the care provided, with a specific focus on end-of-life care considerations. The Canadian Health Care Evaluation Project Questionnaire (CANHELP) [51] *Lite versions* [52] (both patient and family caregiver versions) were selected as they were specifically designed for this purpose. See Table 2 for further description of each instrument.

**Table 2:** Description of selected QOL assessment instruments

Instrument	Construct being measured	Target population	# of items and response scale(s)	Domains measured
ESAS-*r* [39]	Current symptoms	People with life-limiting illness	11 items with a response scale ranging from 0 (no symptom) to 10 (worst possible)	9 items measure individual symptoms, 1 measures wellbeing, and 1 measures a self-identified problem
MQOL-R [49]	Quality of life over the past two days	People at all stages of a life-threatening illness (from diagnosis to cure or death)	14 items (+ 1 global item) with a numerical response scale ranging from 0 to 10.	Physical, Psychological, Existential, and Social
QOLLTI-F [50]	Quality of life over the past two days	Primary family caregiver of patients with life-threatening illness (but developed only with caregivers of cancer patients)	16 items (+ 1 global item) with a numerical response scale ranging from 0 to 10.	Environment, Patient Condition, Caregiver’s Own State, Outlook, Quality of Care, Relationships, and Financial Concerns
CANHELP-lite [52] (patient version)	Satisfaction with end of life care during the past month	Patients with life-limiting illness	20 items with a 5-point response scale ranging from 1 = not at all satisfied to 5 = completely satisfied	Relationship with Doctors, Illness Management, Communication, Decision-Making, Your Well-being, and Overall Satisfaction
CANHELP-lite [52] (family caregiver version)	Satisfaction with end of life care during the past month	Family caregivers of patients with life-limiting illness	21 items with a 5-point response scale ranging from 1 = not at all satisfied to 5 = completely satisfied	Relationship with Doctors, Characteristics of Doctors and Nurses, Illness Management, Communication and Decision-Making, Your Involvement, and Overall Satisfaction

In addition to instrument selection, clinicians highlighted practical hardware design considerations, including the need for tablets to be lightweight, durable, and easy to disinfect between patient uses. Some clinicians suggested that a protective case might be advantageous to counter anticipated patient concerns about tablet fragility, and to provide a better grip in order to facilitate use.

*If I felt that [the tablet] is more protected, I would be more comfortable to take it in my hands and do it. Like people would say, “Oh, my hands are so weak, I can’t like really hold it.” Because of our population, right? We have a majority of our people that are of course end of life, so there is no strength in holding. (Hospital-based nurse).*

A common consideration that clinicians emphasized was that older adults might not be comfortable with technology, leading to a disinclination to use the QPSS. This was not a unanimous belief, however, and some clinicians challenged this common assumption about older adults and technology, such as the following homecare nurse who articulated, “*I do find there are a fair number of seniors who are using their computers. They aren’t dinosaurs, as we might think”.* Indeed, the vast majority of patients who used the QPSS did not have a problem with the technology if they were shown how to use it.

Other desired features focused on software programming of the QPSS system, and referenced the specific needs of older adults living with advancing life-limiting illnesses, and their family caregivers. Clinicians described palliative care patients as frequently fatigued, weak, and cognitively affected by the illness or the use of opioids. Family caregivers were often described as overwhelmed and stressed. Clinicians underlined that as older adults, both patients and family caregivers often have poor eyesight and variable educational background. Finally, clinicians identified that they provide care within a multi-cultural and pluralistic society. Collectively, these considerations resulted in programming recommendations to facilitate visual literacy (large font, single question per screen, audio-interface) and language literacy (faces as visual anchors on scales, assessments available in multiple languages).

From a clinician usability point of view, frequently recommended features included the capacity to interface with electronic medical records, being able to use the system to communicate with other care agencies, and allowing for the printing of assessment results where patient charts are not electronic. Clinicians emphasized the importance of being able to skip questions and save entered data in real time, as well as the ability to enter free text to provide further detail and facilitate interpretation of responses. Rapid feedback on patients’ and family caregivers’ responses to QOL assessment items was identified as an important feature when using the system to inform clinical practice. This is in contrast to previous experiences regarding use of paper-based questionnaires where, in the words of one clinician, “*we never got results so it didn’t guide how you worked*”. A specific desirable feature for reporting results is the ability to graph results over time. In addition, clinicians desired features for tracking treatments and interventions implemented to address identified areas of concern: “*if there could be some way of adding in the information to those graphs that would say, dexamethasone was started here, and then you see the changes, or, this patien*t *had radiation…*”. Clinicians identified this linkage as aiding their ability to determine the efficacy of a specific treatment or intervention. Although there were mixed opinions, some clinicians also commented on the possibility of including cutoff scores to facilitate interpretation with recommendations on how to respond to identified areas of concern.

### Contextual considerations (stage II)

During Stage II, the QPSS was used at least once by 11 clinicians with 27 patients and 8 family caregivers at the palliative care unit, and by 5 clinicians with 11 patients and 5 family caregivers in home care. Despite the enthusiasm in Stage I, once the QPSS prototype was made available, clinicians also expressed significant challenges with integrating the QPSS into their practice. Based on their experiences, as well as the experiences of patients and family caregivers, several overlapping contextual considerations were raised in relation to the technology, use at the point of care, and the health care organization, which are further described below and summarized in Table 3. These contextual considerations encompassed both Stages I and II of the project, although most emerged in Stage II during the initial use of QPSS.

**Table 3:** Summary of results pertaining to the experience of development and use of the QPSS

User group	Themes and corresponding codes
Clinician	*Desirable features for development (Stage I)*
	Ease of use and accessibility of the tablet technology (e.g., accessible storage, long battery life, long plug in cord, accessibility, quality of wireless networks)
	Programming that provides visual accessibility (adjustable text size), appropriate language level, and cultural sensitivity (multiple language options)
	Program flexibility for use in clinical care (e.g., brief assessment, skipping questions, automatic saving of progress, capacity to enter free text details)
	Giving patient and family caregiver control over use of technology (e.g., online interface)
	*Technology context in use (Stage II)*
	Easy to use technology that was predominantly preferable to paper-based assessments
	Concern regarding tablet theft (did not occur)
	Issues with dependability of hospital and home WIFI networks
	Issues with availability of power outlets in the homes
	Downtime during cycles of upgrades in development impacting integration of use into regular practice
	Accessibility of results at the point of care (e.g., interface with electronic medical records, printable results to paper chart, communication with other health agencies, graphing of results to observe trends, tracking of treatments)
	*Point of care context in use (Stage II)*
	Clinician beliefs regarding QPSS limiting its use (e.g., that an older population would not feel comfortable with technology, that technology would negatively impact relational care, that use of QPSS would impose a burden on patients and family caregivers)
	Clinician perception that the QPSS could contribute to more complete assessments or reveal hidden concerns
	Challenge of determining the clinical truth value of numerical scores in the context of high acuity and fluctuating symptoms
	Perceptions of limited clinical follow-up after the use of any standardized assessment instrument
	Better experience when the QPSS is used regularly in order to view trends
	*Health organization context in use (Stage II)*
	Standardized assessments seen as more suited to research than clinical practice
	QPSS perceived to interfere with the use of clinician intuition and clinical judgment
	Busyness and higher acuity in workload creating challenges to integration of QPSS into workflow
	QPSS perceived as administrative surveillance
	Change-fatigue linked to introduction of another new care initiative
Patient and Family Caregiver	*Experiences from stage II*
	Ambivalence using standardized instruments (e.g., concerns of question clarity, recall and truthfulness of responses, and meaning of scores, desired option to enter free text to clarify selected responses)
	Ambivalence using technology (e.g., privacy of information)
	Desired control over the use of technology (e.g., speed of completion, online interface, summary sheet of results to keep for consultation or records)
	QPSS viewed mainly as a data collection tool rather than a tool that could inform their care
	QPSS perceived to help elucidate areas of concern for care
	Fatigue and opiate driven changes in alertness may impact use of QPSS^a^
	QPSS might allow normalization of caregiver areas of concern or underline issues not often discussed (e.g., spiritual or existential concerns) ^b^

^a^ Described by patients only

^b^ Described by family caregivers only

#### Technology context

Patients and family caregivers expressed predominantly positive experiences of the QPSS as a technology. When they had an opportunity to use the QPSS during Stage II of the project, they suggested several additional desirable technological features, including a free text option and the possibility of an embedded audio recorder to provide further context for their numerical scores. As expressed by one family caregiver:

*If there was anything at all, in the two questions that I wasn’t too sure about answering, if I could write underneath and explain the reason… yes, that is what I feel would be very good for the tablet, yes. Emotionally, when you are answering some of the questions, it might be good if that happened – if you could just make that available to answer the question a little bit more fully.*

A few patients and family caregivers expressed some ambivalence regarding the use of electronic devices and standardized instruments to describe how they are feeling, including practical uncertainties about how questions were worded, how to score responses, and the different recall periods of the QOL assessment instruments.

*Well a scale from 1 to 10 isn’t really… it’s like, “Right now, right now, right now.” Right? So it’s kind of like, “Okay, right now. What exactly am I feeling right now?” Because what I’m feeling at 9:00 and what I’m feeling at 9:15 is quite totally different… see the pain, what happens is I take the pain medication and it helps me to get up.*

Other considerations from patients and caregivers included the need for clear assurances about the security of personal information, and having a stylus available (in addition to the touchscreen). A final recommendation included the option for completing assessments independently online (e.g., using a personal device) with the ability to store, retrieve, and share reports of their QOL assessments (also suggested by one of the clinicians). Several patients and caregivers suggested a summary page of answers for their own independent viewing and record keeping.

Clinicians reported logistical, technical, and aesthetic considerations in relation to the QPSS as a technology. Overall, clinicians identified that the QPSS was easy to use, and most preferred it to paper-based assessments. In the hospital setting, clinicians expressed some concern regarding possible theft of tablets. However, clinicians chose to focus on ease of access and therefore tablets were not physically secured, and none were stolen during the project. For home health clinicians, portability issues (battery life, charger cord, ensuring they remembered to bring and remove the prototype from the home setting) were also a significant consideration.

Logistically, prolonged negotiations were required to gain access to the existing wireless hospital network, and the hospital unit and home health office had uneven wireless connectivity (discovered only after the project was in process), which affected consistent functionality of the QPSS. Further, as the QPSS was a prototype, it underwent several development modifications that at times created delays or interruptions in use. To facilitate use in practice, clinicians affirmed needing to integrate the QPSS with existing electronic medical records and to have printing capacity for placing results into patient paper-based charts. Finally, a few clinicians expressed reservations about using the QPSS due to discomfort with technology or concerns that patients and family caregivers may find it “cold.”

#### Point of care context

Clinicians in both hospital and home care voiced important considerations pertaining to the introduction of the QPSS prototype at the point of care. Although the benefits of QOL assessment instruments were recognized, clinicians also expressed discomfort with using standardized instruments for QOL assessments in general, and with older palliative care populations. Some clinicians were concerned about the objective truth-value of numerical scales in the context of QOL assessments. They also expressed concern about the potential burden use may place on patients and family caregivers, and that patients or family caregivers may not understand questions being asked or may not wish to use numerical indicators to assess their QOL. Hospital clinicians, in particular, referenced patient symptom acuity and fluctuation as a barrier to use and a potential threat to the validity of QOL assessment information. Although the details of these considerations varied somewhat based on hospital unit versus home care context, clinicians at both sites also expressed concern regarding how to best integrate the QPSS into existing workflow and workload. This perspective is illustrated in the following quote from a hospital clinician (registered nurse) who commented on QPSS use for family caregiver assessments: “*You know, the reality of things is we are as busy as we are, and acuity is going up. So the reality… I don’t see us being able to do such thing. So I think it’s going to be more onto the social work”.* However, patients and family caregivers did not corroborate most of these concerns in interviews, although one patient did identify that “grogginess” due to pain medication shaped her first experience of using the QPSS. Use of the QOL assessment instruments was usually quite expedient, with the ESAS-R completed by most patients in less than 3 min and the more comprehensive instruments (MQOL-R, QOLLTI-F, and CANHELP-Lite) in less than 15 min per instrument.

Some clinicians framed QPSS use in relation to their existing clinical assessment expertise, assertions that many areas of QOL are already assessed within conversational aspects of daily care, and concern regarding the lack of follow-up on patient and family caregiver assessment results, regardless of modality. At the extreme, several clinicians viewed the routine use of standardized assessment instruments as interfering with their use of intuition, experience, and developing therapeutic relationships.

*I’ve been resistant to use it, to be very honest, because I don’t know how to incorporate it into my practice. I’ve been doing this homecare nursing for 10 years, and three years as a palliative homecare nurse, and it’s really hard to incorporate an electronic tool to what I’m used to – pen and paper writing and following my gut feelings and emotions as it comes up. I’m more in the moment,* versus *trying to look at the data and say, “Oh, it looks like you’ve got some anxiety here. Let’s talk about that,” when that’s already actually incorporated in my daily assessments [when] I see my patients. So a lot of the things that are in these tools, I’ve already incorporated in my own way.*

On the other hand, several clinicians commented favorably about how using the QPSS provided them an opportunity to complete assessments or identify concerns that may not have been otherwise revealed, as exemplified in the following comment from a home care clinician:

*Although we’re looking holistically at what’s going on, sometimes you get just… you don’t see the family members and how they’re doing as much as we do [for] the client, as far as symptoms and stuff go. So it just helps you broaden your understanding of what the big picture is in the house.*

Clinicians also identified greater value in using standardized QOL assessment instruments with patients who are able to complete the assessments regularly; *“When you get someone who can consistently fill out the [assessment instrument] day after day and is orientated to answer it, it gives you a really good perception of what they’re viewing as their issues.”*

Patients and family caregivers offered predominantly positive perspectives regarding the use of the QPSS. However, the QPSS was predominantly viewed as a data collection tool; there was limited recognition regarding its use as part of a clinical assessment to inform the care they received. Nonetheless, several commented on how a QPSS could help to reveal important areas of concern. They also highlighted how use of the QPSS at the point of care enabled privacy and facilitated completion of the assessments at their own pace. One family caregiver added that completing the QPSS helped to normalize the stress he felt in his caregiver role:

*There was one question that asked me how I felt about the way I was communicating with my wife while I was caring for her. And I don’t think anybody’s ever asked me that before. And generally, there is different emotions when you’re caring for things, depending on what physical state she’s in at the moment or what’s been happening prior in the day. Yeah, so I don’t think anybody has ever asked me a question about that before.*

#### Health care organization contexts

Finally, both hospital and home care clinicians discussed QPSS integration in to the context of overarching priorities and considerations of their health care organizations. Clinicians spoke about the benefits of using the QPSS for administrative data collection, and a powerful way to make QOL considerations and experiences of care visible within a health care system increasingly shaped by managerialism and fiscal priorities. At the same time, they also framed use of the QPSS through the lens of administrative surveillance, “fatigue” of never-ending health care initiatives, and skepticism regarding the validity of standardized QOL assessment instruments for use in routine clinical care (rather than for research purposes). Consequently, while some clinicians expressed interest in continuing discretionary use of a more developed QPSS system, they also expressed reservations that mandated use of any QOL assessment instruments, including support systems for their delivery (such as the QPSS), eroded the time for nurses to practice what one clinician called “the art of nursing”. Some clinicians expressed uncertainty as to how assessment results in existing practice are currently aggregated and used, leading to skepticism that assessment results from using the QPSS would benefit resource allocation at local care levels, or increase attention to patients’ and family members’ QOL and health care experiences.

## Discussion

The process of designing and integrating a QPSS in palliative clinical practice is complex and requires extensive consultation with clinicians, administrators, and health care recipients (patients and family caregivers) to ensure that the technology will be useful in meeting the needs of all users. Clinicians valued the opportunity to inform the design and adaptation of a QPSS for their practice setting and provided valuable recommendations regarding hardware and software features. Although patient and family caregiver participants were predominantly enthusiastic, clinicians expressed a mixture of enthusiasm, receptivity, concern, and even resistance regarding the use of electronic systems and standardized QOL assessment instruments. The analyses revealed the importance of paying attention to contextual considerations pertaining to the technology, use at the point of care, and health care organizational contexts. Technical and organizational challenges impacted introduction of a QPSS prototype and included practical constraints regarding integration of the technology within the institutional environment (e.g., access to Wi-Fi) and integration with existing data systems (paper-based chart and electronic medical records). Clinicians further expressed concern with perceived increased workload and a lack of clarity regarding the value and use of QOL assessments in clinical practice.

There are several limitations to our study that need to be taken into account when interpreting the results. It is particularly important to keep in mind that the setting and sampling procedures resulted in limited engagement of clinicians, patients, and family caregivers from different cultural and language backgrounds. In addition, the use QOL assessment instruments to inform routine clinical practice was relatively unfamiliar to most of the clinicians. An exception is the ESAS-*r*, which was mandated in one of the practice settings. Finally, our study focused only on the initial stages of implementation, which involved limited routine use of the QPSS prototype.

The findings from our study complement those of other studies that have examined the implementation of electronic systems for QOL assessment in palliative care. Several studies have focused on the potential benefits of QOL assessments and their effectiveness in enhancing outcomes of care [28, 41]. However, research on the process of implementation is limited. Support for clinicians in implementing QOL assessments has been highlighted in several other studies on the process of introducing QOL assessment instruments in palliative care [53–55]. A literature synthesis by Antunes, Harding, and Higginson regarding the implementation of PROMs in hospital- and community-based palliative care settings specifically highlighted the importance of (a) educational support, (b) consideration of knowledge, skills, and attitudes of individual clinicians, and (c) the presence of a coordinator who can facilitate the implementation process [53]. Studies focusing on other (non-palliative care) populations have similarly revealed the importance of training clinicians to use QOL assessment instruments in their practice [56]. The User’s Guide for Implementing Patient-Reported Outcomes Assessment in Clinical Practice by the International Society for Quality of Life Research provides further useful guidance in addressing methodological and practical decisions regarding the introduction of a QPSS [57]. Our results additionally suggest that guidance is needed for navigating complex and constantly changing health care contexts within which such implementation is to take place.

Our study contributes to this evolving area of knowledge development by conceptualizing implementation of electronic QOL assessment in relation to contextual considerations pertaining to (a) the point of care context, (b) the technology, and (c) the health care organizational contexts. We concur with the findings from previous research that, with respect to point of care contexts, there is a significant need for supporting and educating clinicians to enhance their competence in introducing the use of QOL assessment instruments to patients and family caregivers reviewing QOL assessment data with them, and using QOL assessment data to inform person-centered care planning and shared decision making. Clinicians need both knowledge and skills in using QOL assessment data to: identify areas requiring further in-depth assessment, determine appropriate person-centred interventions in collaboration with patients and family caregivers, and use ongoing QOL assessments as the basis for evaluating the effect of these interventions. Underlying assumptions, such as clinicians’ perceptions of electronic systems and standardized QOL assessment instruments as a threat to relational practice, and older adults not being able to use the technology, also need to be addressed. Indeed, in another study, Krawczyk and Sawatzky describe how the use of an electronic QPSS could facilitate relational aspects of care [58]. Overall, further research and theoretical development is needed to construct and evaluate continuing and basic educational programs focusing on the use of QOL assessment data in clinical practice. The results further point towards the importance of research to better understand mechanisms for successful workflow integration of a QPSS in home- and hospital-based palliative care, including timing of QOL assessments when symptoms are fluctuating. Finally, although not the focus of this manuscript, ongoing research and knowledge translation on psychometric evaluation of QOL assessment instruments is needed to enhance the validity of using QOL assessment information in clinical practice, and to thereby enhance the confidence clinicians have in the accuracy and trustworthiness of QOL assessment scores.

In addition to attending to the point of care context, important considerations regarding the technical context need to be addressed to ensure successful integration of electronic QOL assessments. Our results specifically reveal the importance of establishing clarity about technical and system capacities, prompt resolution of unanticipated technical problems, and integration with existing technologies in the health care system. In addition, we recommend conducting preliminary tests to work out as many technical challenges as possible before involving the entire clinical team. It is further important to ensure that the team is educated about the possibility of technical challenges and the need for ongoing development.

Finally, our results reveal the importance of understanding the health care organization context, including alignment with priorities and purposes regarding current and past use of QOL assessment instruments in the organization. In a related publication, we specifically draw attention to the importance of attending to potential tensions arising from competing priorities across micro-, meso-, and macro-levels of decision making in health care systems [59]. Based on these results, we point to the importance of involving both clinicians and administrators in the planning phase of introducing a QPSS and conducting an environmental scan of current policies, practices, and information technologies within the health care organizations prior to integrating electronic QOL assessment technologies.

## Conclusions

There is much interest and momentum in using electronic systems to integrate QOL assessment instruments in clinical practice as the basis for enhancing person-centered care and shared decision- making. However, the process of designing, integrating and supporting sustained use of electronic QOL assessment systems in palliative clinical practice is complex and requires intentional engagement with health care providers (administrators and clinicians) and recipients (patients and family caregivers) in their initial design and implementation. In addition to seeking input on desired usability features, it is important to understand and respond to contextual considerations pertaining to the technology, use at the point of care, and health care organizational contexts as the basis for enhancing chances of successful integration and sustainability.

## Abbreviations

CANHELP: Canadian Health Care Evaluation Project Questionnaire
ESAS-r: The Edmonton Symptom Assessment System – Revised
KTA: Knowledge To Action
MQOL-R: McGill Quality of Life Questionnaire – Revised
PREM: Patient-Reported Experience Measures
PROM: Patient-Reported Outcome Measures
QOL: Quality Of Life
QOLLTI-F: Quality of Life in Life Threatening Illness – Family Carer Version
QPSS: Quality of Life and Practice Support System
